# Rationing in an era of multiple tight constraints: Is cost-utility analysis still fit for purpose?

**DOI:** 10.1007/s40258-023-00858-w

**Published:** 2024-02-08

**Authors:** Helen Dakin, Apostolos Tsiachristas

**Affiliations:** 1 Health Economics Research Centre, University of Oxford, UK; 2 Nuffield Department of Primary Care Health Sciences, University of Oxford, UK; 3 Department of Psychiatry, University of Oxford, UK

**Keywords:** Health technology assessment, cost-effectiveness analysis, economic evaluation, healthcare resource allocation, prioritization, quality-adjusted life-year (QALY)

## Abstract

Cost-utility analysis may not be sufficient to support reimbursement decisions when the assessed health intervention requires a large proportion of the healthcare budget or when the monetary healthcare budget is not the only resource constraint. Such cases include joint replacement, COVID-19 interventions and settings where all resources are constrained (e.g. post-COVID-19 or in low/middle income countries). Using literature on health technology assessment, rationing and reimbursement in healthcare, we identified seven alternative frameworks for simultaneous decisions about (dis)investment and proposed modifications to deal with multiple resource constraints. These frameworks comprised: constrained optimisation; cost-effectiveness league table; ‘step-in-the-right-direction’ approach; heuristics based on effective gradients; weighted cost-effectiveness ratios; multi-criteria decision analysis (MCDA); and programme budgeting and marginal analysis (PBMA). We used numerical examples to demonstrate how five of these alternative frameworks would operate. The modified frameworks we propose could be used in local commissioning and/or health technology assessment to supplement standard cost-utility analysis for interventions that have large budget impact and/or are subject to additional constraints.

## 1 Introduction

Cost-utility analysis (CUA) is frequently used to assess the value of healthcare interventions and is the recommended method for evaluating drug cost-effectiveness in most Organisation for Economic Co-operation and Development (OECD) countries [1]. Where CUA is used, resource allocation decisions are generally made through a deliberative process taking into account other factors (e.g. equity). Interventions are generally considered cost-effective if their incremental cost-effectiveness ratio (ICER) is below our ceiling ratio or “threshold” [2]. This decision rule would maximise the number of quality-adjusted life-years (QALYs) generated within a given healthcare budget [3] if certain assumptions hold [3, 4].

In this paper, we focus on two such assumptions that, when violated, may make CUA unsuitable for treatment adoption decisions. Firstly, CUA assumes that the intervention in question uses only a small proportion of the healthcare budget and has therefore only a marginal effect on the shadow price of a QALY [3, 5, 6]. Researchers and decision-makers typically assume a constant ceiling ratio [7], but adopting new interventions requires simultaneous disinvestment in other interventions to balance the books, which would gradually reduce our ceiling ratio over time [3, 8]. Lower thresholds based on health opportunity costs have been proposed for high-budget interventions [9]. Secondly, CUA assumes that the monetary healthcare budget is the only resource constraint and ignores non-financial constraints (e.g. staff or beds) [10]. Economic evaluations generally take a long-run view [11] and implicitly assume that resource constraints will be quickly resolved within the timeframe of the decision [10], although constraints often limit their implementation [12].

Constraints may have some flexibility in the medium-term. However, even if most constraints could in theory be resolvable, in practice, many healthcare resource constraints (e.g. staff, equipment, beds, buildings) have persisted for long periods and been experienced (inter)nationally. In the UK, this is reflected in historically-persistent waiting times [13] and staff vacancies [14]. These may arise from an employment monopsony reducing labour prices [2, 10] and difficulties anticipating demand for resources (e.g. consultants or hospitals) that have long lead times.

There are many examples of interventions with large impact on healthcare budgets or where resource constraints cannot be ignored. Firstly, total hip and knee replacement (THR/TKR) costs the NHS around £870 million each year: around 0.6% of the total NHS budget [15–17]. While offering hip and knee replacement to a wider range of patients is likely to be cost-effective [18], the budget impact could be extremely difficult to absorb with current healthcare spending. The numbers of orthopaedic surgeons, operating theatres and ward beds pose additional constraints that have limited increases in the number of operations for many years [18] and waiting lists have increased further since the COVID-19 pandemic [19]. Secondly, government measures to manage the spread of COVID-19 and treat patients cost the UK £310-£410 billion [20]. There were periods with very limited availability of vaccines, test facilities, ventilators, beds and/or staff [21]. Each constraint was short-term (lasting only a few months), but in the acute crisis, policy decisions often had timeframes even shorter than the constraint. Thirdly, there is currently a record backlog of NHS elective surgery and investigations, which is expected to take years to clear [19, 22]. This results from a demand backlog due to the COVID-19 pandemic and supply-side issues (currently 11% of NHS jobs are vacant [14]). Fourthly, in low/middle-income countries where there is limited healthcare budget and shortages of trained staff, medical equipment and drugs, almost any intervention may hit resource constraints and account for a large proportion of healthcare budget [10, 23]. Lifting these constraints is likely to require economic development that could take decades.

Deliberative processes by decision-makers will often take into account resource constraints and budget impact as well as comparing CUA results with an implicit or explicit threshold. However, having a quantitative framework that incorporates resource constraints and the opportunity cost with respect to resources as well as money could help improve decisions.

The aim of this paper was to investigate alternative rationing frameworks that may enable maximisation of health in decisions about interventions that have high budget impact and/or non-monetary resource constraints. We focus on decisions about prioritisation of interventions for health technology assessment, coverage and reimbursement (either at local or national level) in jurisdictions where QALYs or disability-adjusted life-years (DALYs) are considered to be informative measures of health. While decisions or implementation may be constrained by other factors (e.g. political/organisational factors or legal/ethical mandates) [12], we focus on constraints associated with limited healthcare resources.

Section 2 reviews the rationing frameworks and Section 3 demonstrates five of these using a worked example. Section 4 discusses the strengths and weaknesses of the frameworks and how the limitations may be overcome.

## 2 Alternative rationing frameworks

We identified seven rationing frameworks described in the literature that could be adapted to this prioritisation problem and used as an alternative (or complement) to standard CUA (Table 1). We assessed these frameworks against the following criteria:

- provides the largest amount of health within the budget and other resources available;
- supports joint decisions about adoption and disinvestment, weighing the costs and benefits of high-cost interventions and those with additional resource constraints against the current interventions that could be discontinued to free budget/resources;
- makes good decisions on healthcare interventions that vary markedly in size;
- informationally feasible (e.g. it may be impossible to get data on the costs, QALYs and resources for every intervention in our health system);
- easy to explain intuitively, facilitating transparency and deliberation and out-of-the-box thinking – e.g. removing constraints, or saving some money for next year;
- computationally efficient;
- can be used as part of a fair process (e.g. accountability for reasonableness) that is fully transparent, rests on reasons that stakeholders agree on and is revisable in light of new evidence [24].

Cost-benefit analysis was not considered as we take an extra-welfarist approach; cost-benefit analysis also takes no account of either resource constraints or budget constraints except to the extent that individual consumers consider their own resource/budget constraints when giving valuations. Since we focused on health interventions, we also did not consider the approach suggested for multi-sectorial economic evaluation, whereby net benefits are estimated separately for each sector with different ceiling ratios and there is potential or actual compensation between sectors [25].

### 2.1 Constrained optimisation

Constrained optimisation comprises a set of methods for identifying which of a range of alternatives best achieves a stated objective, subject to ≥1 constraint [26]. It can be used to allocate resources across a healthcare system to maximise population health conditional on budget and any other constraints. This integer programming approach has previously been adapted for healthcare [6, 27]. Allocation of healthcare resources can be framed as a “0-1 knapsack problem” [28], where we decide which items to pack in a knapsack to maximise the value, without exceeding a weight constraint or packing >1 of each item.

However, the information requirements and computation time to apply this approach across all healthcare interventions are likely to be infeasible. Constrained optimisation may also fail to identify the best solution when the constraints are flexible and programmes cannot be partially-adopted,^a^ because there may be a solution that gives much higher health with a small change to the constraint [29]. However, ranging analyses are often used to estimate shadow prices: i.e. the change in outcome from a one unit increase in the constraint [30]. If we can carry over some of our underspent budget into next year, it may not be worth using up spare budget on interventions with high ICERs this year.

### 2.2 Cost-effectiveness league tables: a ‘greedy algorithm’

Given one constraint, the cost-effectiveness league table ranks the proposed intervention(s) and interventions that are already part of the healthcare system by ICER and adopts interventions from the lowest ICER upwards until the budget is exhausted [31]. This is equivalent to the greedy algorithm for solving the knapsack problem, where we sort items by bang per buck [32] – the reciprocal of the ICER.

In principle, league tables could simultaneously maximise health subject to both financial and non-financial constraints. We could use the league table to adopt interventions from the lowest ICER upwards until the first constraint is reached, then employ one of several different decision rules to allocate the remaining resources (see Appendix).

### 2.3 ‘Step-in-the-right-direction’ approach

Birch and Gafni proposed that we would better reflect the budget constraint if we adopted interventions only if we can identify a combination of ≥1 current interventions that can be discontinued to free up enough funds to cover the cost of our proposed intervention and which generate fewer QALYs than the proposed intervention [33, 34]. This process would be repeated for other proposed and current interventions. This decision rule has been found to approach the resource allocation that maximises QALYs within the budget as the number of iterations approaches infinity, but take more iterations to converge on the optimal resource allocation than CUA with a ceiling ratio that reflects the least cost-effective current intervention [35]. It works best if past decisions are rational and the treatments currently adopted maximise health from the available budget, but even if this is not the case, it would converge towards the optimal solution if existing interventions are periodically reviewed [35]. It does not necessarily require data on all interventions simultaneously, but would need data on all interventions to converge on the optimal resource allocation.

In principle, this approach could be generalised to multiple constraints simply by identifying ≥1 current intervention(s) for which the total cost *and* total resources are at least as great as the proposed intervention and switching if the proposed intervention(s) has higher QALYs (see Appendix).

However, as with the modified league table, there are many different substitutions that could be made. Whether or not we adopt a proposed intervention will depend on which current intervention(s) it is compared against. Unless there is a decision rule to identify the best substitution, cost-effective interventions may be rejected simply because of an arbitrary choice about which interventions to consider disinvesting in, which would not be acceptable to the public, politicians or pharmaceutical companies.

### 2.4 Heuristics based on effective gradients

Numerous algorithms have been proposed for solving knapsack problems with multiple constraints using “effective gradients”: i.e. the reward for that item divided by the effective capacity (how many copies of that item would theoretically fit in our knapsack if it were used for that item alone) [28]. Heuristics based on aggregate resource use add up effective capacity across all resources (Equation 1), whereas the primal effective capacity heuristic (PECH) takes the minimum effective capacity (Equation 2). If we want to maximise QALYs, and intervention *j* uses *a*_*ij*_ units of resource *i* and ${\bar{b}}_{i}$ is the number of units of resource *i* that are left, the effective gradient is equal to the number of QALYs we would gain by spending all of our available resources on that intervention:

(1) $$Aggregate\;effective\;gradient\;=QALY_{j}\cdot \sum_{i}\;{\bar{b}}_{i}/a_{ij}$$

(2) $$PECH\;effective\;gradient\;=QALY_{j}\cdot min_{i}\left\{{\bar{b}}_{i}/a_{ij}\right\}$$

In principle, we might start with an empty knapsack and adopt the intervention with the highest effective gradient (excluding those where $a_{ij}>{\bar{b}}_{i}$, which require more resources than we have), then recalculate the effective gradients using updated estimates of the remaining budget and resources (*b*_*i*_) and adopt subsequent interventions until the resources are exhausted (see Appendix). This is similar to a league table approach using a modification of the ICER that allows for multiple constraints. There is also no obvious way to identify the best treatments that could be partially adopted. To identify which of our current interventions we should stop doing in order to adopt a new intervention, we also need to go through the whole iterative process of allocating the entire budget, which requires data on all interventions.

### 2.5 Weighted CUA

Van Baal et al [10] proposed assigning additional weight to money spent on healthcare resources that are in limited supply when we estimate ICERs to account for multiple resource constraints. The weights should equal the QALYs gained by increasing spending on the scarce resource by £1, divided by the QALYs gained by increasing the total healthcare budget by £1.

This approach has the advantage that it is a modification of the ICERs that researchers and decision-makers already use and is consistent with existing ceiling ratios.^b^ This approach enabled identification of candidates for disinvestment and those for partial adoption in our worked example. The weighted ICER approach also facilitates evaluating one intervention at a time. While it does not require information on the costs, resource use and QALYs of all current interventions, it does require information on the weights that should be assigned to spending on different resources. These weights can only be calculated if we make assumptions about the rationality of current spending and by identifying situations where decisions are based on resource constraints. Furthermore, whenever we adopt one high-volume intervention, the shadow price of the budget constraint and the shadow price of spending on all of the constrained resources required by that intervention will change. This means that we cannot easily use the weighted ICERs in a league table, because the weights will change as we move down the table.

### 2.6 Multi-criteria decision analysis (MCDA)

MCDA was founded in operations research and has been increasingly applied in the healthcare context by academics and decision-makers [26]. This is because it allows for transparent and systematic trade-offs to be made between multiple, sometimes-conflicting criteria and can support priority-setting decision processes involving multiple stakeholders [37]. It ranks ≥2 alternatives based on their performance on predetermined criteria and incorporates preferences of different stakeholders [38]. In healthcare, it has been used in health technology assessment (risk-benefit analysis), priority setting (e.g. universal health coverage), local decision-making, and research fund allocation [39].

MCDA can be used to prioritise several interventions simultaneously based on a multi-composite maximand (i.e. the total score of each intervention on the selected weighted criteria) that goes beyond QALYs, often including equity and patient experience. MCDA could be adapted to consider multiple constraints. Similar to the league table approach, alternatives could be ranked in descending order of their total performance score and adopted until the first constraint is met. The adoption process is then continued with the use of an additional decision rule on investing the remaining resources until the next constraint is met (see Appendix for more details).

### 2.7 Programme budgeting and marginal analysis (PBMA)

PBMA involves reviewing current resources, activity and spending. The aim is to set objectives for improvement (i.e. health improvement review), identify areas of care (or programme budgets) where the same outcomes could be achieved with fewer resources, and then conduct an appraisal of the added benefits and added costs of an investment or the foregone benefits and cost savings of a disinvestment decision. [40–42]. It therefore explicitly aims to maximise benefits and minimise opportunity costs [41] and seeks to make investments only when there is a candidate for disinvestment that frees up sufficient resources and generates less health, similar to Birch’s step-in-the-right-direction approach [33, 34]. Candidates for investment/disinvestment are ranked using pre-defined criteria similar to those of MCDA [40]. PBMA frequently tabulates and/or takes account of physical resources as well as monetary constraints, although we are not aware of any literature on the deliberative processes or heuristics that decision-makers use to maximise benefits when there are multiple constraints. PBMA is frequently used to allocate resources at a local level in developed countries (often within specific specialties) [43], although it has also been used for national decision-making [44].

## 3 Worked example

To illustrate CUA and five of the alternatives,^c^ we used a hypothetical prioritisation problem allocating resources amongst 23 interventions: 17 hypothetical existing interventions; joint replacement in two patient subgroups who are assumed to already receive the intervention; and four proposed interventions, which comprise expansion of joint replacement for four independent, non-overlapping patient groups (Table 2). The number of joint replacements is limited by the availability operating theatre time and budget. Such constraints are likely to have an even greater impact following the COVID-19 pandemic due to operations cancelled during 2020-22 and increasingly insufficient NHS workforce capacity [14]. UK commissioning groups have historically limited access to joint replacement for patients with poor joint function on the Oxford Hip/Knee Score [45], despite evidence that expanding access would be cost-effective [18].

In our numerical demonstrations, the data on the hypothetical interventions is fictitious while the data for the six joint replacement interventions are informed by published data [18]. We assume a financial budget of £1.4 billion and one non-financial constraint: only 128,000 operating theatre hours are available.^d^ For all treatments, the constrained resource is required in the first year. With the exception of MCDA, we use QALYs as the health outcome measure; however, in principle other maximands or equity weights could be used. We also assumed all interventions to be independent (those for TKR/THR represent different subgroups stratified by Oxford Hip/Knee Score). We compare the resource allocation for each of the frameworks in Table 1, but acknowledge that in practice these analyses will feed into a deliberative process that may take account of additional considerations. The Appendix gives additional methods and results.

### 3.1 Results assuming interventions are indivisible

If interventions must be adopted in full or not at all, all of the approaches considered led to a different set of interventions being adopted. Based on standard CUA, all interventions in Table 2 with an ICER below a static ceiling ratio would be adopted. At a £20,000/QALY ceiling ratio, we would adopt joint replacement for all four proposed subgroups and continue to provide interventions A-I (Table 2). We would therefore overspend our constraints by £35.7 million and 30,000 theatre-hours. Reducing the ceiling ratio to £15,000/QALY would prevent the monetary overspend but we would still exceed the available theatre-hours (Table 3). Standard CUA therefore fails to provide meaningful support in this prioritisation problem, advocating a solution that is infeasible within the available NHS resources and takes no account of non-financial constraints.

Constrained optimisation identified a combination of interventions that generated more QALYs than any other approach and was achievable given the current budget and resources (Table 3). Unlike CUA, THR 21-40, F and TKR 42-43 are not adopted under this approach (despite ICERs <£20,000/QALY), whereas J and L were adopted despite higher ICERs. The modified league table allowing for multiple constraints resulted in fewer QALYs than constrained optimisation regardless of the decision rule used, as did PECH (see Appendix).

ICER weights could be based on local criteria for joint replacement referrals if we assume that these reflect the scarcity of theatre time; using this approach on pre-pandemic data, each £1 spent on theatre time would be given a weight of 5.36 (see Appendix). If interventions are indivisible, the resource allocation suggested by this approach would leave £87 million and >23,000 theatre hours unused.

MCDA was outperformed only by constrained optimisation in terms of generated QALYs, and unused theatre hours. However, MCDA incorporated equity (i.e. the distribution of QALYs across different levels of population vulnerability), which is not included in constrained optimisation.

### 3.2 Results allowing for partial adoption

If interventions are indivisible, any underspend or unused capacity is likely to lead to a reduction in QALYs. The more constraints we have, the more likely it is that ≥1 resource will be incompletely used.^e^ In practice, partial-adoption will often be feasible: e.g. for joint replacement, we could simply set our referral threshold at the Oxford Hip/Knee Score that will ensure that we offer surgery to a feasible number of patients, prioritising those for whom surgery will be most cost-effective [47]. However, fixed costs, indivisible resources (e.g. MRI machines) and equity/practical considerations^f^ may mean that there is some degree of “lumpiness” [49]. In practice, there will not be constant returns to scale: for example, incremental costs, QALYs and cost-effectiveness of joint replacement vary with severity [18] and the shadow price of both the budget and the constrained resource will change as we expand the program to more patients. However, we could deal with this by stratifying the populations for the interventions lying closest to the ceiling ratio and those with highly variable returns to scale, so that we can make independent decisions about whether or not to adopt treatment in non-overlapping subgroups that may have approximately constant returns to scale within each subgroup.

Constrained optimisation and weighted ICERs can readily be extended to identify the optimal proportion of patients having each treatment to maximise QALYs given the constraints (see Appendix). With partial adoption of the interventions lying on each constraint, both of these approaches identify the same resource allocation, which generates more QALYs than any feasible solution identified by the other methods (Table 3).

## 4 Improvements and refinements

Each of the alternative frameworks discussed above has advantages and disadvantages (Table 4). However, there are several ways to overcome some of the limitations of these alternative frameworks and improve their feasibility and efficiency: shortening the league table; removing resource constraints; and temporal effects. The Appendix discusses methods for dealing with mutually-exclusive or interacting interventions.

### 4.1 Shorten the league table

Most of the approaches potentially require reliable, up-to-date information on the costs, QALYs, resource use and patient numbers for all current healthcare interventions, which is not feasible. For example, 1779 different drugs are licenced in the UK [50], plus numerous non-pharmacological interventions.

However, three groups of interventions can be omitted from the set of interventions we use to implement the seven frameworks: current interventions that have lower (weighted) ICERs than the proposed intervention(s); current interventions that must continue, even if they were not cost-effective; and (for most approaches) other interventions that are not currently provided (see Appendix). By shortening the league table to exclude these interventions, there is no need to quantify their costs, resource use or QALYs providing there is reasonable evidence to place them in one of these three groups. This step is similar to the process of choosing a set of meaningful programs and initiatives within PBMA [44] or MCDA [37] and could be based on similar principles. The Appendix discusses modifications to these rules to allow for interactions between interventions.

A shortened league table will require far less data than constrained optimisation or a full league table approach and will help to narrow down the potential candidates for disinvestment in weighted CUA or the ‘step-in-the-right-direction’ approach. It will also be clearer and focus on the key trade-offs.

### 4.2 Buy a bigger knapsack

One way to deal with constraints would be to include strategies in our set of alternatives that relax ≥1 constraint or convert budget into a scarce resource: e.g. transferring resources between departments, training new doctors or building new facilities. These may be subject to additional costs; while these strategies would not directly gain QALYs, they may enable adoption of interventions that do gain QALYs.

For COVID-19, certain constraints were even flexible in the short term: entire hospitals were set up in days, additional medical staff were recruited and vast budgets were reallocated. In joint replacement, we may be able to reallocate budgets relatively quickly and may be able to outsource some operations to private/foreign hospitals, but building new operating theatres and training more surgeons could take years to implement.

In our worked example, adopting all interventions down to G and 10% of H and spending our left-over budget (£14.8 million) on 29,874 hours/year of increased operating theatre capacity would enable us to implement these interventions, accruing 743,320 QALYs: more than any of the feasible solutions in Table 3.

### 4.3 Temporal effects

In standard CUA, we calculate the total present value of the entire lifetime stream of costs and then make a decision about value for money based on today’s threshold, without explicitly considering *when* the costs and resources are required. However, the constraints on our decision-making normally relate to how much resource or money is available in a particular year. Standard CUA cannot account for separate budgets in each year, although mathematical programming techniques have been extended to do this [51]. Patient numbers may also change over time: e.g. we may treat a large group of prevalent patients in the first year, but only incident cohorts thereafter [11]. There may also be short-term inefficiencies when a new technology is introduced. If different interventions require different patterns of resource use or costs over time, it will make it more difficult to identify whether we are in budget. It will also be more difficult to estimate weighted ICERs, since we may need different weights for different years.

For joint replacement, the constrained resources (theatre hours and bed-days) are required immediately, so a single weight can be used and the intervention cannot be delivered if this resource is not available. For unavoidably-constrained resources that are required by a small proportion of patients in later years (e.g. liver transplants in evaluations of hepatitis drugs), the constraint could be dealt with by using a low probability of transplantation, or using discrete event simulation.

Borrowing money to pay back in the future and saving this year’s underspend to use next year are also flexible ways to allow for temporal variations in spending and to expand or contract our budget constraint to maximise QALYs. High-cost interventions are also sometimes implemented more slowly [9, 52]. However, many decision-makers have fixed annual budgets and resources like theatre time or staff time generally cannot be carried forward.

The interventions that expand our knapsack may also introduce temporal effects. For joint replacement, we may need to pay now and get increased operating theatre capacity next year and more surgeons in 10 years’ time. Relaxing constraints may also increase ICERs because of the standard law of supply (e.g. it may cost more to outsource joint replacements to private hospitals). There may also be interventions that increase productivity and therefore decrease ICERs for many interventions: e.g. training nurse practitioners to take over some of the tasks otherwise done by doctors. Morton et al published an algorithm for allocating resources between specific projects and interventions to strengthen the health system [53].

## 5 Conclusions

We examined how seven alternative decision-supporting frameworks perform when making decisions about interventions with high costs and/or resource constraints that cannot be quickly lifted (Table 4). When constraints can be lifted, the costs and benefits of doing so should also be evaluated alongside the intervention that would use that constrained resource.

In our analysis, constrained optimisation and weighted ICERs [10] maximised the health gains from the budget when partial adoption was permitted (Table 3). These approaches gave the same resource allocation in this example, although this is not necessarily true in all cases because the weighted ICERs rely on additional information about the weight for spending on constrained resources.

The weighted ICER approach performed best on our criteria (Table 4), being easy to explain to decision-makers and stakeholders already familiar with CUA and providing a guide for decision-makers to make decisions about partial adoption, purchasing extra resources or saving money for next year. Weighted ICERs also enable decisions to be made one-at-a-time, whereas computerised constrained optimisation algorithms require data on all interventions simultaneously and may be less transparent. Weighted CUA for resource-constrained interventions could also be consistently used alongside standard CUA for non-constrained interventions, whereas other approaches may not be consistent with past decisions. However, spending left-over money on increased operating theatre capacity generated more QALYs: a conclusion that could be reached through a standard league table, weighted CUA league table, PBMA, or MCDA equipped with a league table similar to our example.

There may be particular situations where certain techniques are more appropriate. A league table approach may be more intuitive and transparent than constrained optimisation and show decision-makers the opportunity cost of introducing a new intervention, but may be cognitively challenging when there are multiple constraints. Constrained optimisation involves simultaneous decisions about all interventions, whereas weighted ICERs may facilitate incremental changes to the current resource allocation (e.g. decisions about whether we should adopt a new treatment or disinvest in an existing treatment). Standard league tables performed well when the resource constraints could easily be lifted. PBMA and MCDA may be particularly useful for local decision-making or within a clinical specialty where the number of options is relatively small and where multiple objectives are part of the decision. Some decision-makers find these techniques more intuitive.

Of the approaches discussed, only MCDA and PBMA take account of outcomes other than QALYs, such as equity. However, an MCDA league table will maximise the weighted sum of performance values and will not necessarily maximise health subject to a budget/resource constraint. In principle a composite endpoint, such as net social benefit [6], could be used instead of QALYs in other approaches. Alternatively, MCDA could complement CUA rather than replacing it [37, 54–56].

All of the algorithms discussed require more data than standard CUA. However, much of these data will also be required to inform evidence-based decisions about disinvestment that may currently happen separately from adoption decisions. This may already be collected by local priority-setting committees, similar to the information provided to local commissioners by the Commissioning Support Units and the MCDA-based Prioritisation Framework [57]. Past NICE appraisals and guidelines could also be used to identify current health interventions that are close to the ceiling ratio, as well as information on healthcare costs and QALYs.

Presenting quantities of resources within an impact inventory [58], alongside health and non-health costs and consequences for all economic evaluations would help identify situations where resources may be constrained and provide data that could inform future decisions about investment and disinvestment. This may also encourage policy initiatives that remove constraints.

It is likely that there are many different algorithms used in different disciplines that we have not covered. One approach that has not been considered here is to value resources as the opportunity cost of the second-best alternative rather than expenditure [59]; for constrained resources, the opportunity cost is likely to be higher than the expenditure, but further research would be needed to assess whether this could give similar results to weighted ICERs. More research is also needed on how to account for the timing of resource use and costs when making budget/resource-constrained decisions and on how uncertainty could be dealt with in these frameworks: both around the incremental costs, resource use and QALYs for interventions, and around the budget/resources available.

## Supplementary Material

Appendix

## Figures and Tables

**Table 1:** Overview of alternative rationing frameworks explored in the paper

Rationing framework	Description
0: Cost-utility analysis (CUA)	Adopt proposed intervention if ICER<ceiling ratio
1: Constrained optimisation	Estimate costs, resources and QALYs for every possible combination of interventions; adopt combination with highest QALYs that is within constraints
2: Modified league table	Rank by ICER; adopt from lowest to highest ICER until the first constraint is exhausted; employ a further decision rule to allocate remaining resources. Standard league table is the reciprocal of a greedy algorithm where we rank by value/cost and adopt from highest to lowest
3: Birch & Gafni’s ‘Step-in-the-right-direction’ approach [33, 34]*	Compare QALYs of proposed intervention with combination of ≥1 current intervention(s) with the same or greater cost and resource requirement; substitute if new intervention has greater QALYs
4: Heuristics based on effective gradients: e.g. primal effective capacity heuristic (PECH)	Allocate resources in descending order of the value that they provide relative to how many copies of that item could fit in our remaining resource use
5: Weighted ICER [10]	Assign higher weight to money spent on constrained resources when estimating ICERs
6: Multi-criteria decision analysis (MCDA)	Rank interventions by scores reflecting weighted sum of performance on each criterion; adopt from highest to lowest until the first constraint is exhausted; employ a further decision rule to allocate remaining resources.
7: Programme budgeting and marginal analysis (PBMA)*	Review current activity and spending; identify & rank candidates for investment/expansion/disinvestment based on criteria

* Not analysed quantitatively in numerical examples since there are multiple different possible solutions depending on the existing treatments considered for disinvestment (in the ‘step-in-the-right-direction’ approach) or the decision-makers’ criteria (for PBMA).

Abbreviations: ICER, incremental cost-effectiveness ratio; QALYs, quality-adjusted life-years.

**Table 2:** Interventions and data used in worked examples, presented as a league table

Intervention	Incremental healthcare cost*	QALYs gained*	ICER	Theatre hours*	Patient satisfaction‡	Improves access of vulnerable to care‡	Currently adopted	Cumulative cost	Cumulative theatre hrs	Weighted ICER†	MCDA Total score‡
A	£180,000	174	£1,034	0	Low	Medium	Yes	£180,000	0	£1,034	0.113
B	£80,000	64	£1,250	0	Medium	Low	Yes	£260,000	0	£1,250	0.234
THR <20	£182,080,000	135,863	£1,340	23,538	Low	Medium	Yes	£182,340,000	23,538	£2,090	0.126
TKR <25	£1,108,836,000	585,674	£1,893	74,988	Low	Low	Yes	£1,291,176,000	98,525	£2,448	0.478
THR 21-40	£12,736,000	4,385	£2,904	6,193	Medium	Low	Proposed	£1,303,912,000	104,718	£9,018	0.039
TKR 25-41	£37,510,000	12,151	£3,087	23,870	Medium	Low	Proposed	£1,341,422,000	128,588	£11,591	0.044
C	£4,340,000	1,050	£4,133	6	Medium	Low	Yes	£1,345,762,000	128,594	£4,160	0.039
THR 41-45	£214,000	40	£5,350	100	High	Low	Proposed	£1,345,976,000	128,694	£16,173	0.107
D	£1,860,000	326	£5,714	50	Low	Low	Yes	£1,347,836,000	128,744	£6,379	0.048
E	£4,965,000	690	£7,195	5,000	Medium	Medium	Yes	£1,352,801,000	133,744	£38,563	0.023
F	£23,832,000	2,355	£10,119	24,000	Low	Low	Yes	£1,376,633,000	157,744	£54,234	0.042
TKR 42-43	£332,000	31	£10,710	130	High	Low	Proposed	£1,376,965,000	157,874	£28,864	0.081
G	£2,485,000	207	£12,034	0	High	Low	Yes	£1,379,450,000	157,874	£12,034	0.039
H	£55,250,000	3,000	£18,417	0	Medium	Medium	Yes	£1,434,700,000	157,874	£18,417	0.022
I	£962,500	49	£19,643	60	Low	Low	Yes	£1,435,662,500	157,934	£24,944	0.056
J	£210,000	10	£21,000	0	Medium	Low	Yes	£1,435,872,500	157,934	£21,000	0.110
K	£8,612,500	345	£25,000	0	High	Medium	Yes	£1,444,485,000	157,934	£25,000	0.020
L	£920,000	35	£26,286	0	Medium	Low	Yes	£1,445,405,000	157,934	£26,286	0.052
M	£960,000	33	£29,091	9	High	Low	Yes	£1,446,365,000	157,943	£30,272	0.049
N	£11,397,500	259	£44,091	0	High	Low	No	£1,457,762,500	157,943	£44,091	0.034
O	£16,415,000	343	£47,857	0	Medium	Medium	No	£1,474,177,500	157,943	£47,857	0.021
P	£875,000	10	£87,500	0	Low	Medium	No	£1,475,052,500	157,943	£87,500	0.043
Q	£4,200,000	42	£100,000	0	Low	Medium	No	£1,479,252,500	157,943	£100,000	0.029

Interventions A-Q are hypothetical, while those for total hip replacement (THR) and total knee replacement (TKR) are based on published data [18]. All interventions are independent and are on non-overlapping populations. Costs, QALYs, theatre hours and cost-effectiveness ratios are all assumed to be incremental and compared against “standard care”. The cumulative cost and cumulative theatre hours are estimated by adding the current row to all those that have gone before and represent the total amount of money allocated. Abbreviations: ICER, incremental cost-effectiveness ratio; MCDA, multi-criteria decision analysis; QALY, quality-adjusted life-year; THR <20, total hip replacement for patients with Oxford hip score (OHS) <20; THR 21-40, total hip replacement for patients with OHS of 25-40; THR 41-45, total hip replacement for patients with OHS of 41-45; TKR <25, total knee replacement for patients with Oxford knee score (OKS) <25; TKR 25-41, total knee replacement for patients with OKS of 25-41; TKR 42-43, total knee replacement for patients with OKS of 42-43.

* For the population of England.

† ICER recalculated such that each £1 spent on theatre time is multiplied by 5.36 (1 plus the ratio of the lambda for theatre costs and overall spending – see Appendix). Each hour of theatre time was assumed to cost £992.70.[60]

‡ Multi-criteria decision analysis (MCDA) total [performance] score: see Appendix for methods used to calculate this score; ratings of patient satisfaction and access are vulnerable to care are hypothetical ratings that were used for MCDA, but are not taken into account in other rationing frameworks.

**Table 3:** Comparison of alternative prioritisation frameworks for allocating a budget of £1.4 billion and 128,000 theatre-hours

Intervention	0: CUA (Rc=£15,000)	1: Constrained optimisation		2: Modified league table§		4: PECH	5: Weighted ICER [10]		6: MCDA§	
**Allow partial adoption**	No*	No*	**Yes†**	No*	**Yes†**	No*	No*	**Yes†**	No*	**Yes†**
A	Y	Y	Y	Y	Y	Y	Y	Y	Y	Y
B	Y	Y	Y	Y	Y	Y	Y	Y	Y	Y
THR<20	Y	Y	Y	Y	Y	Y	Y	Y	Y	Y
TKR<25	Y	Y	Y	Y	Y	Y	Y	Y	Y	Y
THR 21-40	Y		Y	Y	Y	Y	Y	Y		
TKR 25-41	Y	Y	97%		97.5%			97%	Y	Y
C	Y	Y	Y	Y		Y	Y	Y		
THR 41-45	Y	Y		Y		Y			Y	Y
D	Y	Y	Y	Y		Y	Y	Y	Y	Y
E	Y	Y		Y		Y				
F	Y									22%
TKR 42-43	Y			Y		Y			Y	Y
G	Y	Y	Y	Y	Y	Y	Y	Y	Y	Y
H		Y	92%	Y	Y			92%		
I		Y		Y		Y			Y	Y
J		Y		Y	Y	Y			Y	Y
K				Y		Y				
L		Y		Y		Y			Y	Y
M				Y		Y			Y	Y
N				Y					Y	Y
O										
P				Y					Y	Y
Q									Y	Y
Total cost (millions)	*£1,379*	£1,400	£1,400	£1,397	£1,398	£1,329	£1,313	£1,400	£1,353	£1,358
Total theatre hrs	*157,874‡*	127,611	128,000	110,073	128,000	110,074	104,774	128,000	122,745	128,000
Total QALYs	746,058	**739,332**	**742,329**	732,243	741,228	728,976	727,742	**742,329**	734,968	735,484

Abbreviations: ICER, incremental cost-effectiveness ratio; MCDA, multi-criteria decision analysis; QALY, quality-adjusted life-year; PECH, primal effective capacity heuristic (heuristic based on effective gradients); QALY; Rc, ceiling ratio per QALY gained; THR <20, total hip replacement for patients with Oxford hip score (OHS) <20; THR 21-40, total hip replacement for patients with OHS of 25-40; THR 41-45, total hip replacement for patients with OHS of 41-45; TKR <25, total knee replacement for patients with Oxford knee score (OKS) <25; TKR 25-41, total knee replacement for patients with OKS of 25-41; TKR 42-43, total knee replacement for patients with OKS of 42-43.

* Including only whole programs: assuming that interventions cannot be subdivided.

† Including partial-adoption of the next most cost-effective intervention for a proportion of patients.

‡ Exceeds the available resources: the solution shown is not feasible.

§ For the modified league table, interventions were assumed to be adopted from lowest to highest ICER, excluding interventions that would push the budget or theatre hours above a constraint; any interventions that would exceed the budget constraint are not adopted, but interventions with higher ICER would be adopted if they are within-budget, but not adopted if they would increase the cost beyond the budget constraint. For MCDA, a similar approach was used but adopting interventions in order of MCDA total score.

**Table 4:** Comparison of frameworks. All approaches could be used as part of a fair, accountable process, so this criterion is not shown here.

Algorithm	Considers >1 constraint	Maximises health with >1 constraint	Inforation required*	Ease of explanation*	Incorporates multiple objectives	Identifies treatments for partial adoption	Pros	Cons
0: CUA	×	×	•	•••••	†	×	Only need data on current treatment	Takes no account of non-budget constraints Assumes λ is static and known
1: Constrained optimisation: comparing all baskets	✓	✓	•••••	•••	†	✓	Works with any number of constraints Gives optimal resource allocation IF constraints are rigid Can set out all of the assumptions in advance	Computationally expensive Need data on costs, QALYs and resource for every treatment Assumes that the constraints are rigid: may be substantial gains from saving budget for next year, or going over budget Black box: likely to miss out-of-the-box solutions
2: Multiple constraint league table	✓	×	•••••	••••	†	✓	Get optimal allocation if only 1 constraint Decision-maker can see potential gains from flexible budgets Allows us to part-adopt last treatment if feasible Simple to explain and interpret	Need data on costs and QALYs for every treatment Multiple solutions when there are multiple constraints May not maximise health subject to multiple constraints
3: Birch & Gafni’s ‘Step-in-the-right-direction’ approach [33, 34]	✓	?	•••	•••	†	×	Decisions can be made one at a time: only needs data on 2-5 treatments at a time	Can easily be adapted for multiple constraints May take a lot of comparisons to converge on the optimal solution: in the meantime, may reject cost-effective treatments
4: Heuristics based on effective gradients	✓	×	•••••	•	†	×	May be more computationally efficient than constrained optimisation	Need data on costs and QALYs for every treatment Could miss best solution More complicated than league table May not facilitate disinvestment decisions
5: Weighted ICER [10]	✓	✓	••	•••	†	✓	Could facilitate a modification of one-at-a-time CUA Maximised QALYs from budget & resources in this example	Need to estimate multiple λ values: may not be sufficient data Allowing for the fact that each λ may change with each adoption decision complicates the analysis
6: Multi-criteria decision analysis (MCDA)	✓	×	••••	••	✓	✓	Forces decision-makers to quantify the trade-offs and relative importance of different decision criteria Incorporates non-QALY benefits	Multiple solutions when there are multiple constraints Data demanding exercise Theoretical axioms may be violated (e.g. overlapping criteria) Selection of MCDA technique may influence the results May not necessarily maximise health gains from the budget
7: Programme budgeting and marginal analysis (PBMA)	✓	×	•••	••	✓	?	Already used in local decision-making Incorporates non-QALY benefits Focuses on minimising opportunity costs and maximising benefits	Cognitively demanding: especially with increasing numbers of interventions Need to select which interventions are considered May not maximise QALY gains from the budget Unclear how decision-makers deal with multiple constraints

* Rating from 0 to 5 (•••••)

† Based on a single maximand; although this would typically be QALYs, an alternative measure that incorporates other objectives in a single value could be used. λ, ceiling ratio.

## Data Availability

The illustrative data used in this paper are given in Table 2.

## Key Points for Decision Makers

- Cost-utility analysis does not explicitly take account of non-financial healthcare resource constraints that may prevent adoption of cost-effective treatments.
- We identified seven alternative frameworks for adoption and disinvestment of healthcare technologies and propose ways that these could be modified to allow for multiple resource constraints.
- The modified frameworks we propose could be used in local commissioning and/or health technology assessment to supplement standard cost-utility analysis for interventions that have large budget impact and/or are subject to additional constraints.

## Declarations

a We define partial adoption as adoption of an intervention for some patients but not for all patients. Since it is sometimes unacceptable to partial adopt treatments due to equity concerns, our worked example initially focusses on the situation where treatments must be adopted in whole or not at all, before exploring a scenario with partial adoption (see Section 3.2).

b This is of particular relevance to the UK, where the Voluntary Scheme for Branded Medicines Pricing and Access stipulates that the standard cost-effectiveness ratio for the National Institute for Health and Clinical Excellence (NICE) will not be changed for the duration of the voluntary scheme [36]. For treatments that do not have a major impact on constrained non-monetary resource (which may include most oral medications that do not require intensive monitoring), decision-making with weighted ICERs will be the same as that for standard one-at-a-time CUA.

c PBMA and the ‘step-in-the-right-direction’ approach are not analysed quantitatively in numerical examples since there are multiple different possible solutions depending on the existing treatments considered for disinvestment (in the ‘step-in-the-right-direction’ approach) or the decision-makers’ criteria (for PBMA).

d Costs, QALYs and theatre time in Table 2 are incremental compared with the next best alternative. The budget and theatre-time constraints are therefore assumed to exclude the cost and resources that would be required if we did not adopt any of the interventions in Table 2 and simply provided their comparator, “standard care”.

e High-volume interventions have a disproportionately high chance of being the last intervention adopted because they take up such a large proportion of the budget/resources and are more likely to use it all up [46]. Furthermore, the benefits of part-implementation will be largest for interventions with large total QALY gains.

f For example, postcode prescribing may be unacceptable and clear evidence on differences in clinical effectiveness/safety may be needed to justify rationing by age, ethnicity or sex [48].

## Code availability

Further details of the methods used are given in the Appendix. The Stata code and spreadsheets used to implement MCDA, effective gradients and constrained optimisation are available from the authors on request.
