# Supplementary material for: Rationing in an era of multiple tight constraints: Is cost-utility analysis still fit for purpose?
Source: Appl Health Econ Health Policy. Author manuscript; Available in PMC 2024 May 1. (PMC7615833; doi:10.1007/s40258-023-00858-w)
Supplement: Appendix [file EMS193192-supplement-Appendix.docx]

**Appendix**

Appendix to “Rationing in an era of tight and multiple resource constraints: Is cost-utility analysis still fit for purpose?” *Applied Health Economics and Health Policy*.

Helen Dakin DPhil,^1^ and Apostolos Tsiachristas PhD

^1^Health Economics Research Centre, University of Oxford, UK. [helen.dakin@dph.ox.ac.uk](mailto:helen.dakin@dph.ox.ac.uk)

**Funding statement:** HD and AT are financially supported in part by the National Institute for Health and Care Research (NIHR) Oxford Biomedical Research Centre (BRC). AT would also like to acknowledge financial support from the NIHR Applied Research Collaboration Oxford and Thames Valleyand the NIHR Oxford Health BRC.The views and opinions expressed therein are those of the authors and do not necessarily reflect those of the NIHR, NHS or the Department of Health.The funding agreement ensured the authors’ independence in designing the study, interpreting the data, writing, and publishing the report.

**Conflict-of-interest disclosure:** The authors have no competing interests to declare that are relevant to the content of this article.

**Additional methods and results of quantitative analyses**

***Constrained optimisation***

We implemented constrained optimisation using the Solver add-in to Excel 2016. The analysis allowing only full adoption of programmes used the evolutionary solving method to maximise the number of QALYs by varying the proportion of full implementation for each of the 23 interventions, subject to three constraints:

- The proportion of each intervention that is implemented must be a binary integer ≤1 and ≥0.
- The sum of the product of the proportion adopted multiplied by the theatre hours across all interventions must be less than our assumed constraint of 128,000 hours.
- The sum of the product of the proportion adopted multiplied by the cost across all interventions must be less than our assumed budget of £1.4 billion.

This analysis was also repeated in Stata Release 15 (College Station, TX: StataCorp LLC) by first identifying all 2^23^ possible baskets of the health interventions in Table 2, and then identifying the basket with highest QALYs of those that cost ≤£1.4 billion and ≤128,000 theatre-hours. This analysis gave the same resource allocation decisions as Solver but took 2.5 minutes to run, whereas Solver gave results almost immediately.

The analysis allowing for partial adoption used the Simplex LP solving method^[[1]](#footnote-2)^ to maximise the number of QALYs by varying the proportion of full implementation for each of the 23 interventions, subject to three constraints:

- The proportion of each intervention that is implemented must be ≤1 and ≥0.
- The sum of the product of the proportion adopted multiplied by the theatre hours across all interventions must be less than 128,000 hours.
- The sum of the product of the proportion adopted multiplied by the cost across all interventions must be less than £1.4 billion.

The spreadsheet and Stata code used for these analyses are available from the authors on request.

***Modified league table***

In a standard league table with a budget of £1.4 billion, we could in theory adopt down to intervention G(Table 3), gaining 743,009 QALYs.However, based on our assumed constraint of 128,000 operating theatre hours, we could not feasibly implement interventions TKR 25-41, C, THR 41-45, D, E, F and TKR 42-43 at the same time.

We explored several different decision rules that we could use to allocate resources subject to two constraints using a modified league table. These all involve adopting interventions from lowest to highest ICER until the first constraint is reached, but differ in the rules that are used to allocate resources after that point. These decision rules are illustrated in Table A1.

1. Adopting interventions from lowest to highest ICER until the first constraint is reached, and adopt no further treatments. This leaves large amounts of budget and theatre hours unused.
2. Adopting interventions from lowest to highest ICER, excluding interventions that would push the budget or theatre hours above a constraint; any interventions that would exceed the budget constraint (e.g. intervention O) are not adopted, but interventions with higher ICER would be adopted if they are within-budget (e.g. P), but not adopted if they would increase the cost beyond the budget constraint (e.g. Q). In this case, we would adopt A, B, THR<20, TKR<25 and THR 25-40, but would not have enough theatre-hours to adopt TKR 25-41; we would then consider the remaining interventions in ascending order of ICER, excluding those requiring theatre hours. This leads to us rejecting high-volume interventions with low ICERs in preference to those with lower resource/budget requirements and higher ICERs: e.g. we would adopt P, not TKR 25-41. It also leads to us adopting TKR for mild symptoms (scores 42-43), but not moderate symptoms (TKR 25-41), even though the health gains and net benefit is greater for moderate symptoms. This could be considered clinically unjustifiable.
   1. As (2) but not adopting TKR 42-43 on equity grounds.
   2. As (2) but stopping adoption when the budget constraint is first reached and not considering any further treatments with higher ICER even if they are within budget. For example, intervention O would increase spending beyond the budget constraint, so this treatment is not adopted and (unlike (2)), no further interventions with higher ICER (P and Q) are considered.
   3. As (2b) but not adopting TKR 42-43 on equity grounds.
3. As (1) but also partially adopting the first treatment that is beyond any constraint. In this case, we would adopt TKR 25-41 for 97.5% of patients, which would fully exhaust the theatre hours, but leave budget unspent.
4. Adopting interventions from lowest to highest ICER,until the first constraint is reached, then partially-adopting the first treatment that is beyond this constraint (in this case TKR 25-41), then adopting treatments in ascending order of ICER until the budget constraint is exhausted, skipping any treatments that require the resource that has already been used up.

**Table A1:**Comparison of alternative decision rules for allocating a budget of £1.4 billion and 128,000 theatre-hours using a modified league table

| **Treatment** | **Decision rule number (see text above)** | | | | | | |
| --- | --- | --- | --- | --- | --- | --- | --- |
|  | **1** | **2** | **2a** | **2b** | **2c** | **3*** | **4*** |
| A | yes | yes | yes | yes | yes | yes | yes |
| B | yes | yes | yes | yes | yes | yes | yes |
| THR <20 | yes | yes | yes | yes | yes | yes | yes |
| TKR <25 | yes | yes | yes | yes | yes | yes | yes |
| THR 25-40 | yes | yes | yes | yes | yes | yes | yes |
| TKR 25-41 | no | no | no | no | no | 97.5% | 97.5% |
| C | no | yes | yes | yes | yes | no | no |
| THR 41-45 | no | yes | yes | yes | yes | no | no |
| D | no | yes | yes | yes | yes | no | no |
| E | no | yes | yes | yes | yes | no | no |
| F | no | no | no | no | no | no | no |
| TKR 42-43 | no | yes | no | yes | no | no | no |
| G | no | yes | yes | yes | yes | no | yes |
| H | no | yes | yes | yes | yes | no | yes |
| I | no | yes | yes | yes | yes | no | no |
| J | no | yes | yes | yes | yes | no | yes |
| K | no | yes | yes | yes | yes | no | no |
| L | no | yes | yes | yes | yes | no | no |
| M | no | yes | yes | yes | yes | no | no |
| N | no | yes | yes | yes | yes | no | no |
| O | no | no | no | no | no | no | no |
| P | no | yes | yes | no | no | no | no |
| Q | no | no | no | no | no | no | no |
| Cost (thousands) | £1,303,912 | £1,397,296 | £1,396,964 | £1,396,421 | £1,396,089 | £1,340,499 | £1,398,444 |
| Theatre hours used | 104,718 | 110,073 | 109,943 | 110,073 | 109,943 | 128,000 | 128,000 |
| QALYs | 726,160 | 732,243 | 732,212 | 732,233 | 732,202 | 738,012 | 741,228 |

***Step-in-the-right-direction approach***

The results of the step-in-the-right-direction approach will depend on the order that interventions are considered and which existing interventions are compared against the new intervention. We therefore do not evaluate a set of fully worked results of this approach.

One approach is to considerate new interventions one-at-a-time, starting with the intervention that has the lowest ICER (THR 25-40). We could then consider existing interventions, starting with those with the highest ICER. We could disinvest in those current interventions that have a combined cost and combined theatre days that are at least as large as the proposed intervention and generate fewer QALYs. We might disinvest in interventions L and M and at least partially disinvest in F to free up sufficient budget and theatre hours to balance THR 25-40. Having adopted THR 25-40, we would consider TKR 25-41.

***Heuristics based on effective gradients : Primal effective capacity heuristic (PECH)***

PECH was implemented manually in Microsoft Excel by iterating the following steps:

- Estimating the remaining healthcare budget and theatre hours after all previous interventions have been adopted.
- Estimating equation (2) for each intervention.
- Adopting the intervention with the highest PECH effective gradient.
- Deciding not to adopt any interventions with an effective gradient below 1 (which require more resources or money than we have left).
- Repeating steps 1-4 for any interventions for which decisions have not yet been made.

The spreadsheet used for this analysis is available from the authors on request.

***Weighted CUA***

Past commissioning decisions could be used to estimate the weight that we should place on spending on theatre time.Many clinical commissioning groups (CCGs) limit joint replacement referrals for patients with Oxford hip/knee scores above 18-24 [1]. If we ignore the constrained resources, this criterion would be consistent with commissioners using a ceiling ratio of around £5000/QALY[2].However, CCGs may have been accurately reflecting the scarcity of theatre time when they restricted access to joint replacement for patients with Oxford hip/knee scores >18-24. For this illustration, we estimated ICER weights assuming that a threshold Oxford hip/knee score of 24 reflected the resource constraint for theatre hours.

In a recent study, the incremental cost of THR compared with no THR was £4,747 for patients with an OHS of 24 (averaged over all age/sex groups) and the incremental QALY gain was 2.10[3]. Since the Payment by Results tariff used to estimate the cost of surgery does not separate out the cost of theatre time, we assumed that each operation lasted an average of 65.79 minutes[4] and valued theatre time in orthopaedics at £993/hour[5]. This suggests that the shadow price of spending on theatre time is 0.00183 QALYs per £1 spent (an ICER of £545 per QALY gained), whereas the shadow price of total healthcare spending is 0.000443 per £1 spent (an ICER of £2,256/QALY gained). The weight assigned to spending on theatre time equals 1 plus the ratio of the two lambdas – in this case 1+0.00183/0.000443=5.36. We applied the same figure to both THR and TKR.

However, this figure is very dependent on the assumptions we make about the duration of surgery, which is decreasing over time and may vary between patient groups. The method we used also gives misleading results when the incremental cost of the constrained resource is larger than the total incremental cost (which could easily happen if an initial investment in a scarce resource results in downstream savings).

Applying the weight of 5.36 to all spending on theatre time gives modified ICERs (Table 2, penultimate column) that can be used in the same way as those from conventional CUA to identify which of the proposed joint replacement interventions should be adopted at a £20,000/QALY ceiling ratio and which of the existing interventions should be discontinued. If we assume that these weights are constant, we might consider adopting the proposed intervention that has the lowest weighted ICER (THR 25-40) and then disinvest in current interventions in descending order of weighted ICER until we are within the budget and within thesurgical theatres constraint (thereby discontinuing interventions E, F and H-M; Table 3). However, if interventions are indivisible, we would have £77 million and >23,000 theatre hours unused.

If we allow partial adoption, we would adopt TKR 25-41 for 97% of patients (until the resource constraint was exhausted) and H for 92% of patients (until the budget constraint was exhausted). As this example illustrates, it is only (in this case H and TKR 25-41) for which we may need to assume divisibility and constant returns to scale if we are to fully spend the budget.[6]

***Multi-criteria decision analysis (MCDA)***

There are many methods for performing MCDA and their pros and cons for their application in healthcare are well-documented[7]. In this example, we used multi-attribute value theory (MAVT), one of the most frequently used method of MCDA, to assess the 23 alternatives in terms of their performance on health, patient satisfaction, and equity and determine which of them can be provided within the budget (£1.4 billion) and operating theatre capacity (128,000 hours; Table 2).

In our example, performance scores were standardised using:

$S_{jc}=\frac{x_{jc}}{\left( \sum x_{jc}^{2} \right)^{1/2}}$ (A1)

where$S_{jc}$ $S_{\mathrm{ij}}$ is the standardisation of the performance value $x$of the *j*$i$^th^ alternative against the $j$*c*^th^ criterion (i.e. healthcare costs, QALYs gained, patient satisfaction, improved access of vulnerable to care, Table 2). For costs, where the highest values are the least preferred, the reciprocal of the performance values ($1/x_{jc}$) was used in (3). After standardisation, the performance values have a range between 0 (least preferred) and 1 (most preferred).

We then assigned hypothetical weights to each criterion to reflect the relative importance of each criterion. There are several methods to obtain these weights but their application is outside the scope of this paper[7, 8]. We used the hypothetical weights: 0.25 for healthcare cost, 0.45 for QALYs, 0.10 for patient satisfaction, 0.20 for improves access of vulnerable to care. Complying with MCDA manuals, the hypothetical weights sum to 1[7]. We then used a linear additive model to combine the standardised performance values with the criteria weights to estimate total scores, using $T_{j}=\sum_{c=1}^{n} S_{jc}\times w_{c}$, where $T_{j}$ is the total score for alternative $i$ $j$ and $w_{c}$ is the weight for criterion $j$ $c$.

The alternatives can be ranked in descending order based on total performance score and, similar to a league table approach, alternatives can be adopted until the first constraint is met (i.e. theatre hours). As was the case for league tables based on ICERs, we need an additional heuristic to decide how to allocate the left-over budget/resources.In this case, alternatives G, N, and Q were adopted (in descending order of total performance score) to exhaust the budget as they required no theatre hours. However, like the modified league table, this led to TKR being offered to patients with mild symptoms (Oxford Knee Scores 42-43) but not those with moderate symptoms (Oxford Knee Scores 25-41).

Other MCDA approaches could have been used in this hypothetical example[9], including the multiple criteria multiple constraint (MC^2^) level linear programming that would provide an efficient (or non-dominated) solution based on acceptable and feasible trade-offs between multiple criteria and constraints[10, 11]. However, the selection of the MCDA method should be well justified as it may result in different prioritisation and thorough efforts should be paid to avoid violating the theoretical axioms of the selected MCDA method [12].

**Additional detail on shortened league tables and interactions**

***Methods for shortening the league table (independent treatments)***

As discussed in Section 4.1, three categories of intervention could be omitted from a shortened league table to simplify the analysis and reduce the amount of data required to inform decisions. We give additional examples of these here:

1. Existing interventions that have lower ICERs (and, where applicable, lower weighted ICERs) than the proposed intervention(s). It would clearly not be efficient to disinvest in these highly cost-effective interventions to introduce a new intervention that is worse value for money. There are only four of these in Table 2 (A, B THR <20, and THR 21-40), but could be thousands in reality. Assessing whether interventions are *likely* to have lower ICERs would require a lower standard of evidence than quantifying the costs, QALYs, resource use.
2. Current interventions that would be inappropriate to disinvest in, even if they were not cost-effective. Equity considerations and public preferences may mean that certain services have additional value over and above their QALYs gains. In a crisis (e.g. COVID-19 or a short-term period of budget cuts), it may also be inefficient to cut services that we need to continue in order to have the option of doing them in the future or those that cannot be stopped and started up again in a short space of time (e.g. capital expenses, like hospitals or MRI machines).
3. Interventions that are not currently provided (other than the intervention(s) under consideration) may be excluded from the ‘step-in-the-right-direction’ and weighted ICER approaches, where we can consider each of the proposed interventions in turn, in the context of existing interventions (ignoring other proposed interventions). Although constrained optimisation may propose adoption of interventions with higher ICERs in order to use up the budget, this approach will not be efficient if we can leave budget unspent to use next year. Weighted ICERs will always be at least as high as unweighted ICERs, so interventions that have already been shown to have high unweighted ICERs will not be considered cost-effective in the weighted ICER approach.

In this shortened table, the budget constraint will comprise the total cost and total resources across the currently-adopted interventions that are shown in the shortened table. Because it is assumed that all other interventions will remain the same, the value of the total budget and how it is allocated across other interventions is not relevant.

***Impact of interactions between interventions and mutually exclusive sets***

In the worked example, all treatments are independent:i.e. adopting one treatment has no impact on the incremental costs, theatre hours or QALYs of other interventions. In practice, there are many mechanisms by which this will not be the case in practice and some interventions may be literally mutually-exclusive and cannot be adopted together [13]. In some cases, the impact of interactions on costs, QALYs and resources may be negligible, in which case we may be able to treat the interventions as though they were independent. In other cases, we may be able to mitigate the effect of interactions by stratifying the population and making separate decisions for each subgroup[13] (as is done here for joint replacement to allow for the fact that costs and QALYs vary markedly with Oxford Knee/Hip scores).

If there are interactions between two or more existing treatments that are likely to change decisions about which treatments are adopted even if we stratify the population, it will be necessary to compare different combinations of these interacting treatments incrementally as a set of mutually exclusive treatments[13]: e.g. no treatment, A, B and A+B. Similarly, if there are interactions between an existing treatment and a proposed treatment or between two proposed treatments that are likely to change decisions about which treatments are adopted even if we stratify the population, we could also consider combinations of these treatments as a mutually exclusive set in our league table: e.g. no treatment, proposed only, existing only, and both.

For such sets of mutually-exclusive interventions, we would normally use the following standard rules for decision-making[14, 15]. Firstly, we would exclude any combinations within the mutually-exclusive set that are dominated (though either strong or extended dominance). Secondly, the non-dominated combinations from all of the sets would be listed together in the league table in descending order of their ICER relative to their next best non-dominated alternative. The incremental costs, QALYs and (in our case resource use) for each intervention would be estimated relative to the next best non-dominated alternative. If we were using the standard league table approach and decided to adopt the second intervention in the set (e.g. A), this would mean that we would replace the first intervention (e.g. no treatment) in the set with the second intervention (A) and therefore accruethe incremental cost of A versus no treatment (plus the cost of no treatment, which is excluded from both the budget constraint or the total costs this example). However, this process becomes more complex for standard league tables when we have multiple constraints as treatments may be dominated with respect to resources, but not with respect to cost (or vice versa).Using the weighted ICER approach, we could deal with this problem by estimating weighted ICERs comparing different combinations of the interacting interventions, excluding those combinations that are dominated based on their weighted ICER and evaluating non-dominated combinations incrementally based on weighted ICERs.

Within constrained optimisation, different combinations of interacting treatments could be included in the set of interventions. Constrained optimisation requires the costs, QALYs and resource use for each intervention to represent the total, rather than the incremental. Additional constraints could be applied to ensure that only one of a set of mutually exclusive treatments is adopted.

***Methods for shortening the league table when interventions are not independent***

Interactions between interventions will also affect the three groups of interventions that can be omitted from a shortened league table:

1. Existing treatments that have (weighted) ICERs that are expected to be lower than that of the proposed intervention *regardless of what other interventions are implemented at the same time* can be excluded from the shortened league table.
2. If it is not possible to disinvest in an intervention, this is unlikely to be affected by interactions unless the availability of the proposed intervention affects the constraints preventing the existing treatment from being discontinued. For example, if a treatment currently cannot be stopped because patients would otherwise get no treatment, but the proposed treatment would provide an alternative intervention for that group, it may be appropriate to consider switching from the old treatment to the new treatment.
3. Existing treatments that are not currently provided can be excluded from the ‘step-in-the-right-direction’ and weighted ICER approaches *unless adoption of the new treatment would materially change their costs, QALYs or resource use*.

If the costs, resource use and/or QALYs associated with the proposed treatment will differ depending on what other treatments are adopted but the conclusions for the existing treatment are unlikely to be materially affected, we could do a sequential analysis, whereby the costs, resources and QALYs for the proposed treatment are based on those that would apply if treatments in groups 1 and 2 (from the list above) were adopted and treatments in group 3 were not adopted.

However, if there are non-ignorable interactions that could change the conclusions for both the proposed treatment and the existing treatment and cannot be mitigated by stratifying the population, we would need to make a joint decision between mutually-exclusive combinations of the two treatments (e.g. neither, existing only, proposed only and proposed plus existing) and include all non-dominated combinations within the set in our shortened league table.

**References for Appendix**

1. The Royal College of Surgeons of England. Is access to surgery a postcode lottery? 2014 [Available from: <https://www.rcseng.ac.uk/news-and-events/media-centre/press-releases/many-ccgs-are-ignoring-clinical-evidence-in-their-surgical-commissioning-policies/>. Accessed 8 Nov 2016.

2. Dakin HA, Eibich P, Gray A, Smith J, Barker KL, Beard D, et al. Who gets referred for knee or hip replacement? A theoretical model of the potential impact of evidence-based referral thresholds using data from a retrospective review of clinic records from an English musculoskeletal referral hub. BMJ Open. 2020;10:e028915. <https://doi.org/10.1136/bmjopen-2019-028915>

3. Dakin H, Eibich P, Beard D, Gray A, Price A. The use of patient-reported outcome measures to guide referral for hip and knee arthroplasty. Part 2: A cost-effectiveness analysis. Bone Joint J. 2020;102-B:950-8. <https://doi.org/10.1302/0301-620X.102B7.BJJ-2019-0105.R2>

4. Chaudhry FA, Ismail SZ, Davis ET. A new system of computer-assisted navigation leading to reduction in operating time in uncemented total hip replacement in a matched population. Eur J Orthop Surg Traumatol. 2018;28:645-8. <https://doi.org/10.1007/s00590-018-2133-y>

5. ISD Scotland. R142X: Theatre direct cost per hour, by speciality: April 2014 - March 2015 24th November 2015 [Available from: <https://www.isdscotland.org/Health-Topics/Finance/Costs/File-Listings-2015.asp>. Accessed 6 November 2020.

6. Johannesson M, Weinstein MC. On the decision rules of cost-effectiveness analysis. J Health Econ. 1993;12:459-67.

7. Marsh K, M I, Thokala P, Baltussen R, Boysen M, Kalo Z, et al. Multiple Criteria Decision Analysis for Health Care Decision Making--Emerging Good Practices: Report 2 of the ISPOR MCDA Emerging Good Practices Task Force. Value Health. 2016;19:125-37. <https://doi.org/10.1016/j.jval.2015.12.016>

8. Thokala P, Devlin N, Marsh K, Baltussen R, Boysen M, Kalo Z, et al. Multiple Criteria Decision Analysis for Health Care Decision Making--An Introduction: Report 1 of the ISPOR MCDA Emerging Good Practices Task Force. Value Health. 2016;19:1-13. <https://doi.org/10.1016/j.jval.2015.12.003>

9. Marsh K, Lanitis T, Neasham D, Orfanos P, Caro J. Assessing the value of healthcare interventions using multi-criteria decision analysis: a review of the literature. Pharmacoeconomics. 2014;32:345-65. <https://doi.org/10.1007/s40273-014-0135-0>

10. Chen DD, Zhong YH, Liao YX, Li LN. Review of Multiple Criteria and Multiple Constraint-level Linear Programming. Procedia Comput Sci. 2013;17:158-65. <https://doi.org/10.1016/j.procs.2013.05.022>

11. Zhong YH, Chen DD, Cui GF, Jia YL, Li LN, Liao YX. The Theory of Multi-criteria and Multiple Constraint-level Linear Programming for Oilfield Development. Procedia Comput Sci. 2013;17:141-8. <https://doi.org/10.1016/j.procs.2013.05.020>

12. Marsh KD, Sculpher M, Caro JJ, Tervonen T. The Use of MCDA in HTA: Great Potential, but More Effort Needed. Value Health. 2018;21:394-7. <https://doi.org/10.1016/j.jval.2017.10.001>

13. Dakin H, Gray A. Decision Making for Healthcare Resource Allocation: Joint v. Separate Decisions on Interacting Interventions. Med Decis Making. 2018;38:476-86. <https://doi.org/10.1177/0272989X18758018>

14. Karlsson G, Johannesson M. The decision rules of cost-effectiveness analysis. Pharmacoeconomics. 1996;9:113-20.

15. Drummond MF, Sculpher MJ, Claxton K, Stoddart GL, Torrance GW. Methods for the Economic Evaluation of Health Care Programmes. 4th ed. New York: Oxford University Press; 2015.

1. The Simplex LP algorithm built into Solver solves linear programming problems by moving iteratively along the edges of the feasible region to maximise or minimise a linear objective function. [↑](#footnote-ref-2)
